# Ion channel P2X7 receptor in the progression of cancer

**DOI:** 10.3389/fonc.2023.1297775

**Published:** 2024-01-11

**Authors:** Guang-ping Zhang, Jun-xiang Liao, Yi-yi Liu, Fu-qi Zhu, Hui-jin Huang, Wen-jun Zhang

**Affiliations:** ^1^ The Second Affiliated Hospital, Nanchang University, Nanchang, Jiangxi, China; ^2^ Department of Critical Medicine, Ganzhou people’s Hospital, Ganzhou, Jiangxi, China; ^3^ Department of Rehabilitation Medicine, the Second Affiliated Hospital, Nanchang University, Nanchang, Jiangxi, China

**Keywords:** P2X7, tumor microenvironment, predictor, treatment, macrophages

## Abstract

P2X7 receptor (P2X7) is a non-selective and ATP-sensitive ligand-gated cation channel. Studies have confirmed that it is expressed in a variety of cells and correlates with their function, frequently in immune cells and tumor cells. We found increased expression of this receptor in many tumor cells, and it has a role in tumor survival and progression. In immune cells, upregulation of the receptor has a double effect on tumor suppression as well as tumor promotion. This review describes the structure of P2X7 and its role in the tumor microenvironment and presents possible mechanisms of P2X7 in tumor invasion and metastasis. Understanding the potential of P2X7 for tumor treatment, we also present several therapeutic agents targeting P2X7 and their mechanisms of action. In conclusion, the study of P2X7 is an important guideline for the use of clinical tumor therapy and may be able to provide a new idea for tumor treatment, but considering the complexity of the biological effects of P2X7, the drugs should be used with caution in clinical practice.

## Introduction

The pathogenesis of tumors is complex and involves many factors including genetic changes and tumor microenvironmental changes (such as tumor-associated macrophages, T lymphocytes, and tumor cells) and molecular substances (such as ATP, cytokines, chemokines, growth factors, hypoxia, acidosis, and extracellular matrix). Tumor microenvironment means that the occurrence, growth, and metastasis of tumor are closely related to the internal and external environment of tumor cells. It not only includes the structure, function, and metabolism of tumor tissue but is also related to the internal environment of tumor cells. Tumor cells can change and maintain their own survival and development conditions and promote tumor growth and development through autocrine and paracrine (1, 2). Tumor growth and metastasis remain the main causes of tumor treatment failure (3). Therefore, it is of great importance to explore tumor-related targeting molecules. Although molecular substances associated with tumor pathological progression have been unearthed, more molecular bases need to be explored to target tumor prevention and treatment.

The P2X7 is an ion channel type receptor, and ATP is its natural activator. The presence of sufficient ATP in the tumor microenvironment activates P2X7, and P2X7 activation mediates the opening of membrane ion channels (with an inward flow of sodium and calcium ions and outward flow of potassium ions), mediating intracellular signals and thus regulating tumor cell progression (4, 5). In addition, P2X7 activation can also mediate immune cell activity and inflammatory response to indirectly regulate the growth, migration, and metastasis of tumor cells (6). Tumor growth and metastatic spread were significantly accelerated in P2X7 gene-deficient mice. Intratumor IL-1β and vascular endothelial growth factor release were significantly reduced, and inflammatory cell infiltration was almost completely absent (7). However, the use of P2X7 antagonists may inhibit tumor progression. P2X7 antagonists (A438079 and AZD9056) antagonize P2X7 activity and inhibit the growth, metastasis, and epithelial–mesenchymal transition (EMT) formation of colorectal cancer cells (8). Significantly, P2X7 activation also inhibits tumor progression, which is related to the structure and function of the P2X7 (9). Under the condition of continuous action of activators (such as ATP and BzATP) or sharp exposure to the high concentration of activators, the configuration of P2X7 changed, and the ion channel gradually opened into large membrane pores, resulting in changes in cell membrane fluidity and configuration, leading to cell edema, apoptosis, and death (2, 4). Heat therapy (40°C) was found to enhance the extracellular ATP (eATP)-mediated killing of colon cancer cells. The mechanism is that a high temperature-induced increase in membrane fluidity enhances P2X7 function and pore opening and promotes apoptosis by modulating downstream AKT/PRAS40/mTOR signaling events (9). These studies have revealed the important role of P2X7 in regulating tumor progression; therefore, we explored the potential association between P2X7 and different tumors, focusing on the role of P2X7 on gastric cancer progression and the pharmacological properties of P2X7 as potential molecular targets for targeting tumors.

## Structural characteristics of P2X7

**Figure 1:**
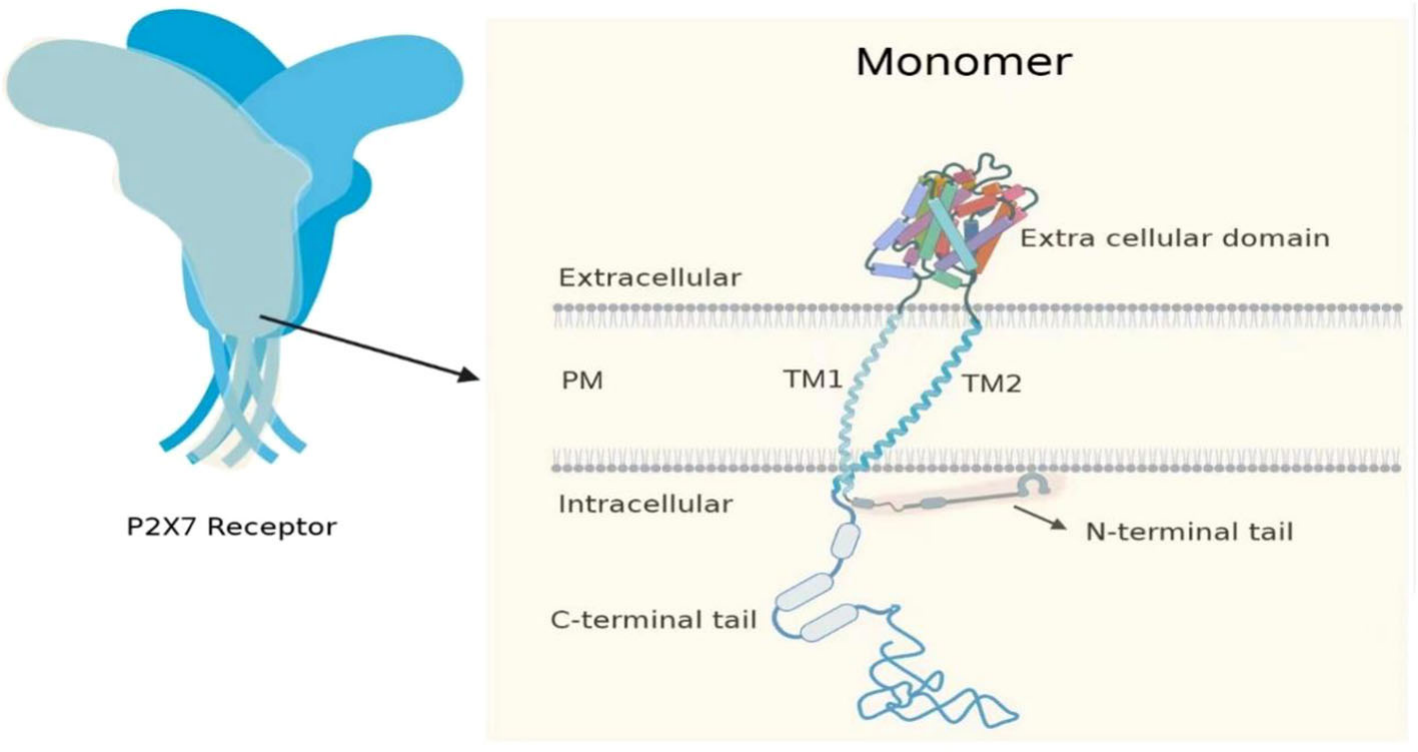
P2X7 consists of three monomers. The P2X7 monomer has a short intracellular N-terminal residue (26 aa), a bulky extracellular domain (282 aa), two transmembrane helices (approximately 24 aa each), and a long cytoplasmic carboxy-terminal tail (239 aa).

The P2X7 is non-selective and sensitive to ATP (10). The gene that encodes the P2X7 in humans is located on the long arm of chromosome 12 at the 12q24.31 locus, which is centromeric and closer to the P2X4 locus (11). The location of P2X7 in mice is at 62.50 cm on chromosome 5. P2X7 gene encodes a protein comprising 595 amino acids (known as the P2X7 subunit or monomer), which forms a trimeric complex to construct the functional P2X7. The P2X7 monomer consists of a short intracellular N-terminal residue of 26 amino acids, a bulky extracellular domain of 282 amino acids, two transmembrane helices each approximately 24 amino acids in length, and a long cytoplasmic carboxy-terminal tail of 239 amino acids (12). The N-terminal is short, and it has a role in regulating calcium influx (13–15). The long C-terminal cytoplasmic tail of 245 aa(aa 350 to 595) surrounds the second transmembrane domain and serves as a weight, playing a role in regulating the opening of macropores (12, 16–18). The initial part of the C-terminal tail consists of a region rich in cysteine, which undergoes palmitoylation on five residues (C362, C363, C374, C377, and S360). This process plays a crucial role in the transportation of the receptor to the cell membrane and in preventing receptor desensitization (18, 19). Two transmembrane domains (TM1 and TM2) have the function of regulating ion channel opening. The trans-membrane domains of TM1 and TM2 in the P2X7 of humans are composed of residues T28-S47 and N332-L354 (12, 20). The P2X family consists of seven members that can assemble into homo- and hetero-trimers at the cell membrane, thanks to the presence of an extracellular domain with an ATP binding site (21, 22) (**Figure 1**). The P2X family members can be differentiated by their varying affinities to ATP, which range from nanomolar levels for P2X1 and P2X3; to low micromolar levels for P2X2, P2X4, and P2X5; to hundreds of micromolar levels for P2X7. They also differ in their rate of channel desensitization, with milliseconds for P2X1 and P2X3; seconds for P2X2, P2X4, and P2X5; and no observable desensitization for P2X7 (21–27). The biphasic response to ATP stimulation is a distinguishing characteristic of P2X7. When ATP induced activation for a relatively short period (2 s or less), P2X7 opens a channel that allows the influx of Na^+^ and Ca^2+^ ions and the efflux of K^+^ ions. However, when ATP stimulation is prolonged (4 s or more), it results in the formation of a larger pore that allows molecules of <900 Da to pass through. Recent reports have demonstrated that ATP stimulation of P2X7 can rapidly open an NMDG+-permeable macropore, without gradual enlargement (28, 29).

P2X7 activation in various cell types, including macrophages, lymphocytes (such as T lymphocytes and B lymphocytes), glial cells (microglia and astrocytes), and tumor cells (including most human tumor cells such as breast cancer, gastric cancer, colorectal cancer, and lung cancer cells), triggers the activation of associated signaling pathways like MAPK, JNK, and ATK. This, in turn, stimulates the release of inflammatory cytokines such as IL-1β, TNF-a, and chemokine ligand 3 while also promoting apoptosis and cell death (30). P2X7 triggers AMPK activation in microglia and macrophages through Ca/CaMKK- and reactive oxygen species (ROS)-dependent mechanisms in nerve cells and immune cells (31). Activation of P2X7 in tumor cells induces the expression of IL-6, IL-8, and MCP-1 through a Ca^2+^-dependent mechanism (32).

P2X7 is found in a variety of animals and has a similar structure, and this structure leads to different functions in different cells. It is widely distributed and plays a significant role that suffices it to show its research significance.

## The relationship between the P2X7 and tumor microenvironment

**Figure 2:**
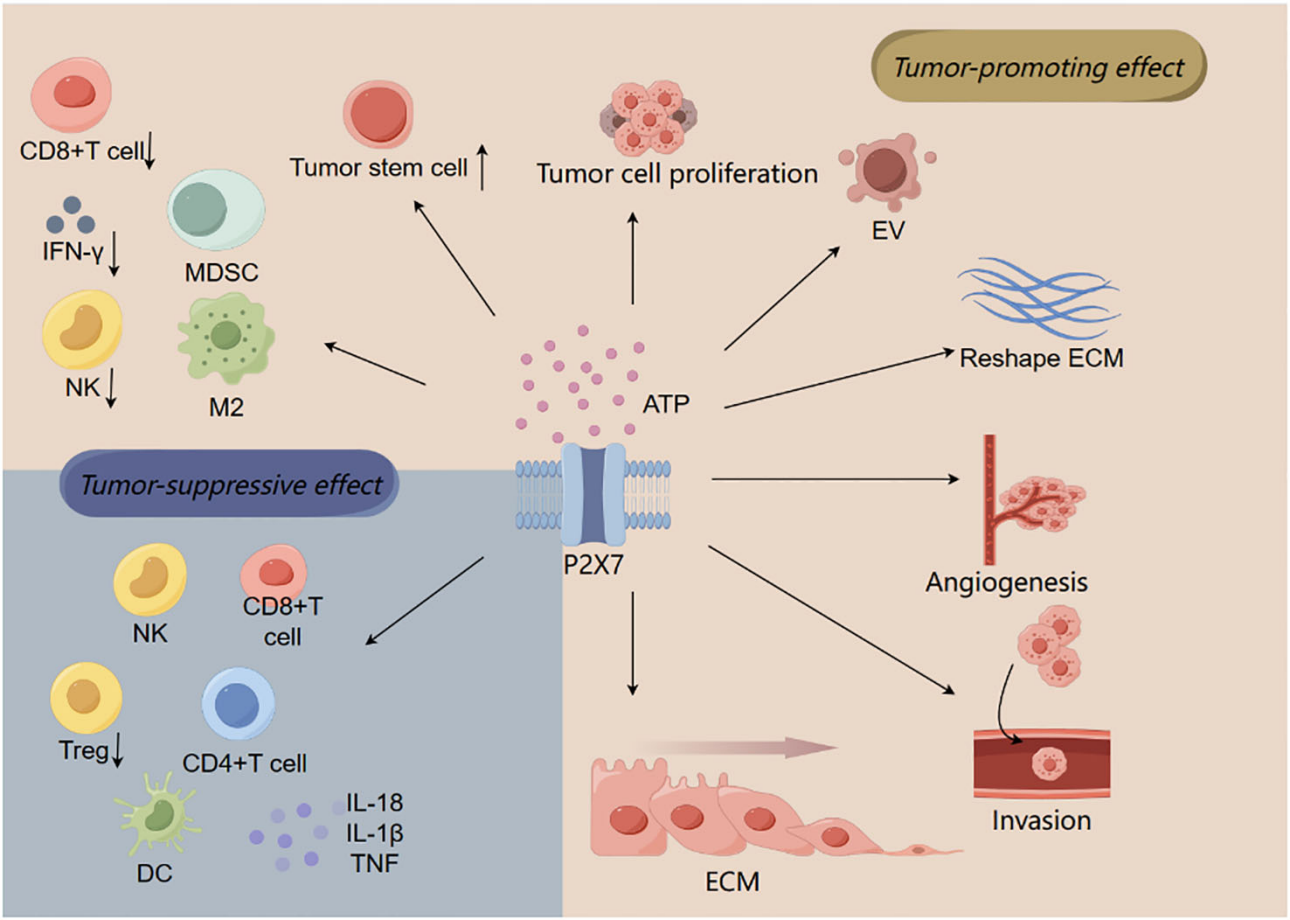
The relationship between P2X7 and tumor microenvironment. ATP activates P2X7 to promote tumor cell migration, invasion, and proliferation by regulating tumor-associated macrophage changes (M2) and T lymphocytes (such as inducing CD8+ T cell death), resulting in immunosuppressive effect. In addition to these effects that contribute to tumor progression, P2X7 can also produce antitumor effects, such as promoting the activation and proliferation of immune cells (CD4+ T cells, CD8+ T cells, and NK cells), increasing the production of cytokines (such as TNF, IL-18, and IL-1β), and reducing the number of Tregs.

The tumor microenvironment (TME) includes immune cells (T cells, NK cells, B cells, tumor-associated macrophages, tumor-associated neutrophils, and dendritic cells (DCs)), stromal cells (tumor-associated fibroblasts), vasculature, extracellular matrix, and ATP. Tumor cells interact with immune cells and stromal cells to alter TME components to promote their growth and metastasis (33–35) (**Figure 2**).

Different studies have shown that P2X7 is increased in tumor cells, such as upregulation of P2X7 in breast cancer (36) and terminal lung cancer (37). Upregulated P2X7 may be associated with their survival and metastasis. In normal tissues, eATP levels are small (concentrations of approximately 10–100 nmol/L) and significantly increased in tissue injury, inflammation, hypoxia, ischemia, and tumor microenvironment. The eATP can originate from passive leakages (cell death), activation of immune cells, or active release via vesicles, where eATP is an inflammatory trigger, but under extracellular nuclease hydrolysis, the product adenosine is an important immunosuppressant (38, 39).

Various immune cells play an important role not only in tumor suppression but also in tumor promotion. Tumor-associated macrophages (TAMs) are usually divided into two types, M1 and M2, which have opposite activities: M1 can exert antitumor effects through direct-mediated cytotoxicity and antibody-dependent cell-mediated cytotoxicity (ADCC), while M2 contributes to tumor growth and spread through anti-inflammation, immunosuppression, promotion of vascular proliferation, and tissue repair (40). P2X7 can mediate TAM proliferation, differentiation, and apoptosis and plays an important role in innate and intrinsic immunity. P2X7 is highly expressed in TAM (41). P2X7 deficiency impairs the “M2-like polarization” of TAM by downregulating the phosphorylation of STAT6 and IRF4, promotes T-cell mobilization, and reverses M2-like TAM polarization by reducing tumor cell proliferation and angiogenesis, thus limiting the progression of uratan-induced lung cancer (41). P2X7 stimulated by eATP enhances extracellular cGAMP transport to macrophages and activates STING to exert antitumor effects (42). Triggering the eATP–P2X7–inflammasome–IL18 axis has been shown to reduce the number of macrophages within tumors, enhance intratumor T-cell effector function, overcome anti-PD-1 resistance, and potentially enhance the effectiveness of adoptive T-cell metastasis (43).

Among T cells, CD8+ T cells are the main performers of tumor immunosurveillance, mediating apoptotic necrosis of tumor cells mainly through TCR recognition of MHCI–tumor peptide complexes, the release of perforin and granzyme, or through the FasL pathway. In addition, CD4+ T cells also play a role in TME. Activated CD4+ T cells can secrete IL-2 or support pro-inflammatory dendritic cells to activate CD8+ T cells to exert antitumor effects (44–46). For T cells, P2X7 can affect the migration and movement of T cells and play an immune infiltrative role, but also it has a role in the differentiation of effector T, in which the growth and function of memory CD8+ T cells cannot be achieved without P2X7 (47). This indicated that P2X7 was associated with antitumor immunity. However, this is the physiological concentration of ATP, but it is significantly increased in TME, in which ATP-mediated cytotoxicity can lead to T cell death. Although Treg also expresses high P2X7, the extracellular nucleotidases CD39 and CD73 also increase in parallel relative to P2X7, and they can degrade ATP into adenosine, which helps Treg to escape the cytotoxic effect of P2X7 and act as immunosuppression, helping tumor cells to immune evade (48). Studies have shown that the increase of tumor necrosis in CD39-deficient mice is related to the expression of P2X7 (49). P2X7+CD8+ T-cell subsets decreased in human lung adenocarcinoma. P2X7+CD8+ T cells are sensitive to Art1-mediated ADP ribosylation and NICD, and this sensitivity increases after blocking ADP ribosomal cyclase CD38, which degrades NAD+ (50). In mouse non-small cell lung cancer and melanoma models, genetic and antibody-mediated Art1 inhibition slowed tumor growth in a CD8+ T cell-dependent manner, which was related to the increased infiltration of activated P2X7+CD8+ T cells in the tumor (50). Tumor-specific P2X7−/−CD8+ T cells showed mitochondrial maintenance and impaired function but showed no signs of failure in the early stage of antitumor response (51). However, with the increase in tumor burden, the relative frequency of P2X7-deficient CD8+ T cells decreased, which was related to decreased proliferation, increased apoptosis, and mitochondrial dysfunction (51). Studies have shown that transient stimulation of P2X7 with ATP analog BzATP can enhance the control of B16 melanoma by CD8+ T cells (51).

Hypoxia is an important feature in TME associated with tumor progression, aggressiveness, and metastasis. TME also has vascular proliferation to satisfy the oxygen demand associated with tumor cell proliferation. Vascular proliferation benefits from the secretion of epidermal growth factor and VEGF by endothelial cells (33, 34). Simulated hypoxia may enhance the activity of P2X7 by relying on HIF-1α and PIP2 activation pathway (52). Hypoxia can increase the expression of advanced glycation end-product receptor RAGE and P2X7 and promote the invasive ability of tumor cells (53). In neuroblastoma cells, P2X7 activation promotes HIF-1α level and VEGF secretion by activating PI3K/AKT/GSK3β/MYCN and HIF-1α/VEGF3 signaling pathways, upregulates the expression of HIF-1α and VEGF, and increases intracellular glycogen reserve (54). This suggests that P2X7 promotes the proliferation of blood vessels to deliver nutrients to tumor cells and promote the spread of tumor cells. Meanwhile, P2X7 has a trophic effect on tumor cells by upregulating GLUT glucose transporter protein and related enzymes in glycolysis and inhibiting pyruvate dehydrogenase to promote glycogen accumulation and cell growth (5).

In addition, the extracellular matrix can be remodeled by tumor cells and other cells to form a microenvironment suitable for tumorigenesis (34, 55). Overexpression of MMPs, including MMP2 and MMP13, promotes tumor progression mainly through degradation of extracellular matrix components (56, 57). P2X7 can regulate the changes in extracellular matrix composition and promote tumor migration and invasion. Continuously activated P2X7 triggers the release of active MMP2, thereby preventing ion channels and macroporous reactions by cleavage of MMP2-dependent receptors (58). ATP5B overexpression increases extracellular ATP levels through intracellular ATP secretion, activates FAK/AKT/MMP2 signaling, and promotes the proliferation, migration, and invasion of tumor cells. The activation of downstream pathways induced by ATP5B is induced by plasma membrane P2X7 (59). ATP activating P2X7 can downregulate the protein level of E-cadherin, upregulate the production of MMP-13, and promote the invasion and migration of breast cancer (60). Studies have shown that P2X7 activation can downregulate the protein expression of E-cadherin, upregulate the mRNA and concentration of MMP-2, and promote the migration and invasion of tumors (61).

P2X7 is a cytotoxic receptor that, upon short-term activation by ATP, opens a non-selective cation channel that promotes Ca^2+^ influx, while chronically high concentrations of P2X7 open to form an osmotic macropore, which is detrimental to tumor cells, but it can also express a non-macroporous functional P2X7 heterodimer (nfP2X7) that escapes this injury (5, 38). In contrast, in the rat glioma model C6, Ca^2+^ influx and macropore formation were observed, but the results were enhanced cell proliferation and migration and reduced apoptosis, which could be related to the resistance of rat gliomas to ATP-mediated cytotoxicity (62). In lung cancer cells, treatment of A549 (human lung adenocarcinoma cell) and H1299 (non-small cell lung carcinoma cell) with eATP revealed the formation of F-actin-rich filamentous foot-like protrusions on the plasma membrane, suggesting that P2X7 may be associated with cell spreading (63). The above conjecture was further demonstrated in an experimental mouse model of metastatic melanoma cells, in which 90% of B16-F10 melanoma cells expressing P2X7 showed significant pulmonary metastases in mice, while the number of mice showing pulmonary metastases was significantly reduced after antagonizing P2X7 with A740003 (64). However, P2X7 is also widely expressed in immune cells and plays an important role in TME. In TME, ATP induced P2X7 to activate DCs to assemble pro-inflammatory vesicles IL-1β (47). It has also been demonstrated that activation of P2X7 can induce the conversion of Treg into pro-inflammatory IL-17-secreting cells (65), playing a tumor-suppressive role.

In conclusion, P2X7, an adenosinergic receptor, is important in tumor development, affecting the infiltration of immune cells, the entire purinergic system of TME, and extracellular nucleases. Moreover, the interaction between P2X7 and Panx1, P2X4, CASP1, NLRP3, and so on is also important. Therefore, some drugs targeting P2X7 can be effective in treating tumors but should be used with caution given their complex biological effects (66).

## The high and low expression of P2X7 in tumor

P2X7 is expressed in many cells, including tumor cells. Studies have shown that the expression of P2X7 in human gastric cancer is higher than that in normal gastric tissue. Abnormal high expression of P2X7 was associated with larger tumor size, higher T stage, and lymphatic metastasis (67).

**Figure 3:**
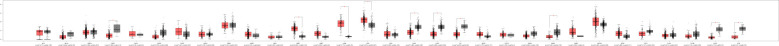
Expression of P2X7 receptor in different types of tumors. This figure illustrates the differential expression levels of P2X7 in different kinds of tumors, including adenoid cystic carcinoma, bladder urothelial carcinoma, breast invasive carcinoma, cervical squamous cell carcinoma, endocervical adenocarcinoma, cholangiocarcinoma, colon adenocarcinoma, lymphoid neoplasm diffuse large B-cell lymphoma, esophageal carcinoma, glioblastoma multiforme, head and neck squamous cell carcinoma, kidney chromophobe, kidney renal clear cell carcinoma, kidney renal papillary cell carcinoma, acute myeloid leukemia, brain lower-grade glioma, liver hepatocellular carcinoma, lung adenocarcinoma, lung squamous cell carcinoma, ovarian serous cystadenocarcinoma, pancreatic adenocarcinoma, pheochromocytoma, paraganglioma, prostate adenocarcinoma, rectum adenocarcinoma, sarcoma, skin cutaneous melanoma, stomach adenocarcinoma, testicular germ cell tumors, thyroid carcinoma, thymoma, uterine corpus endometrial carcinoma, and uterine carcinosarcoma. Red represents tumor samples, and gray represents normal samples. The horizontal axis represents different tumor types, and the vertical axis represents TPM, reflecting the associated expression levels. *p < 0.01 indicates statistical significance.

In the microenvironment where tumors grow, high levels of extracellular ATP activate P2X7. By inducing cells to secrete IL-6, substance P, and other cytokines, P2X7 is involved in cell proliferation and tumor metastasis. Studies have confirmed that P2X7 is highly expressed in some tumors, such as prostate (68), breast cancer and skin cancer (69, 70), neuroblastoma (71), thyroid cancer (72), and B-cell chronic lymphocytic leukemia (73) (**Figure 3**). Some tumors are low in expression, such as endometrial epithelial carcinoma (74). In most studies, it has been reported that there is a trend of upregulation of P2X7 in tumor tissues but no analysis of functional activity.

In human thyroid papillary carcinoma, P2X7 stimulation triggers the release of the growth factor IL-6 in thyroid cells, while in human neuroblastoma, this receptor supports the release of the growth factor substance P in these cancer cells. Neuroblastoma cells show another interesting feature: although P2X7 is fully functional and induces the formation of the typical “large conductively porous”, ATP has a little cytotoxic effect, as if neuroblastoma cells have learned to separate pore-formation from the death-inducing pathway. This “death escape” mechanism has not been studied, but it may be related to uncoupling with caspases because neuroblastoma cells do not exhibit caspase-3 activation in response to P2X7 stimulation compared to many other non-tumor cell types.

Cancerous epithelial cells degrade P2X7 mRNA by activating an unstable domain located in the 3′UTR of P2X7. The present results may be important for our understanding of the role of P2X7 in the development of endometrial cancer. The background theorem is that defective apoptosis leads to cancer. The results show that the degradation degree of P2X7 gene 3′UTR in cancer cells is higher than that in normal cells, indicating that the 3′UTR region of P2X7 gene contains unstable sites, and the rapid degradation of P2X7 mRNA in cancer cells was the result of activation in the 3′UTR region (74). Different expression levels of P2X7 lead to different apoptosis events (75–77). P2X7 is low-expressed not only in carcinoma endometrial cells but also in complex hyperplasia cells with atypia, similar to the previous findings in cervical/cervical dysplasia cells (78).

P2X7 has been reported to be expressed in a variety of cell types including monocytes/macrophages, dendritic cells, mast cells, mesangial cells, and microglia (79–83). The triggering of P2X7 may stimulate cell proliferation or induce apoptosis, depending on the level of activation. Some malignant tumors express higher P2X7 levels; the concentration of ATP in tumor stroma is several times higher than in healthy tissue, in principle enough to activate the P2X7.

It is now clear that P2X7 not only promotes tumor growth but also inhibits tumor growth. Tumor growth inhibition appears to be primarily, if not exclusively, due to promoting (or even allowing) DC/cancer cell interactions, stimulating cytokine release, promoting chemotaxis, and inflammatory cell infiltration into the tumor. The different roles of P2X7 in promoting inflammation (on the host side) and stimulating proliferation (on the tumor side) may explain why P2X7 blocking may promote rather than slow tumor progression in different experimental models (84).

## Invasion and metastasis associated with P2X7

Tumor mortality is primarily caused by the proliferation of cancer cells and their ability to metastasize to other organs. As previously described, different studies have confirmed that P2X7, a protein receptor, is often overexpressed in tumor cells and may play a role in cancer cell invasion and metastasis.

P2X7 plays a crucial role in the progression of ATP affecting tumor metastasis. ATP, as a source of cellular energy, promotes cell motility, enhances extracellular matrix degradation, and induces cell migration (85). It is worth noting that there is a difference in ATP levels between tumor and non-tumor cells in the extracellular microenvironment, with tumor cells containing more ATP (86). Metastasis is a complex process, and the metastasis pathways of different tumors in which P2X7 is involved are often various.

**Figure 4:**
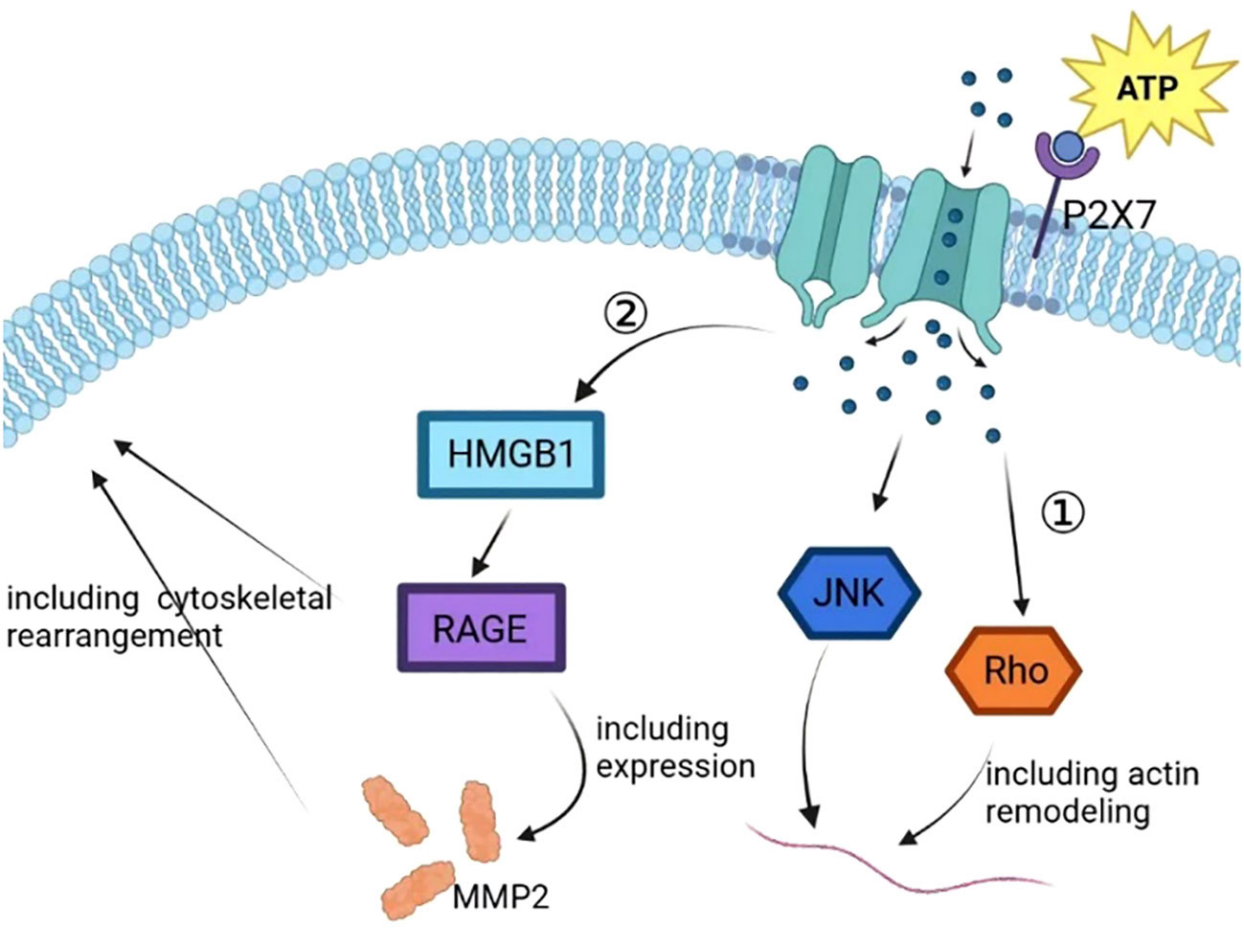
In the process of tumor progression, the ATP released by cells enters the extracellular matrix, resulting in a sharp increase in the concentration of ATP in the microenvironment and activating the P2X7 on the tumor cell membrane. P2X7 activation leads to self-configuration changes, leading to the opening of ion channels on the membrane to mediate ion flow, further activating JNK and Rho, upregulating the expression of VEGF, and promoting tumor vascular growth. P2X7 activation can also promote tumor progression by activating HMGB1/RAGE signal, regulating EMT/metastasis-related gene MMP2 expression, and inducing cytoskeleton rearrangement.

**Figure 5:**
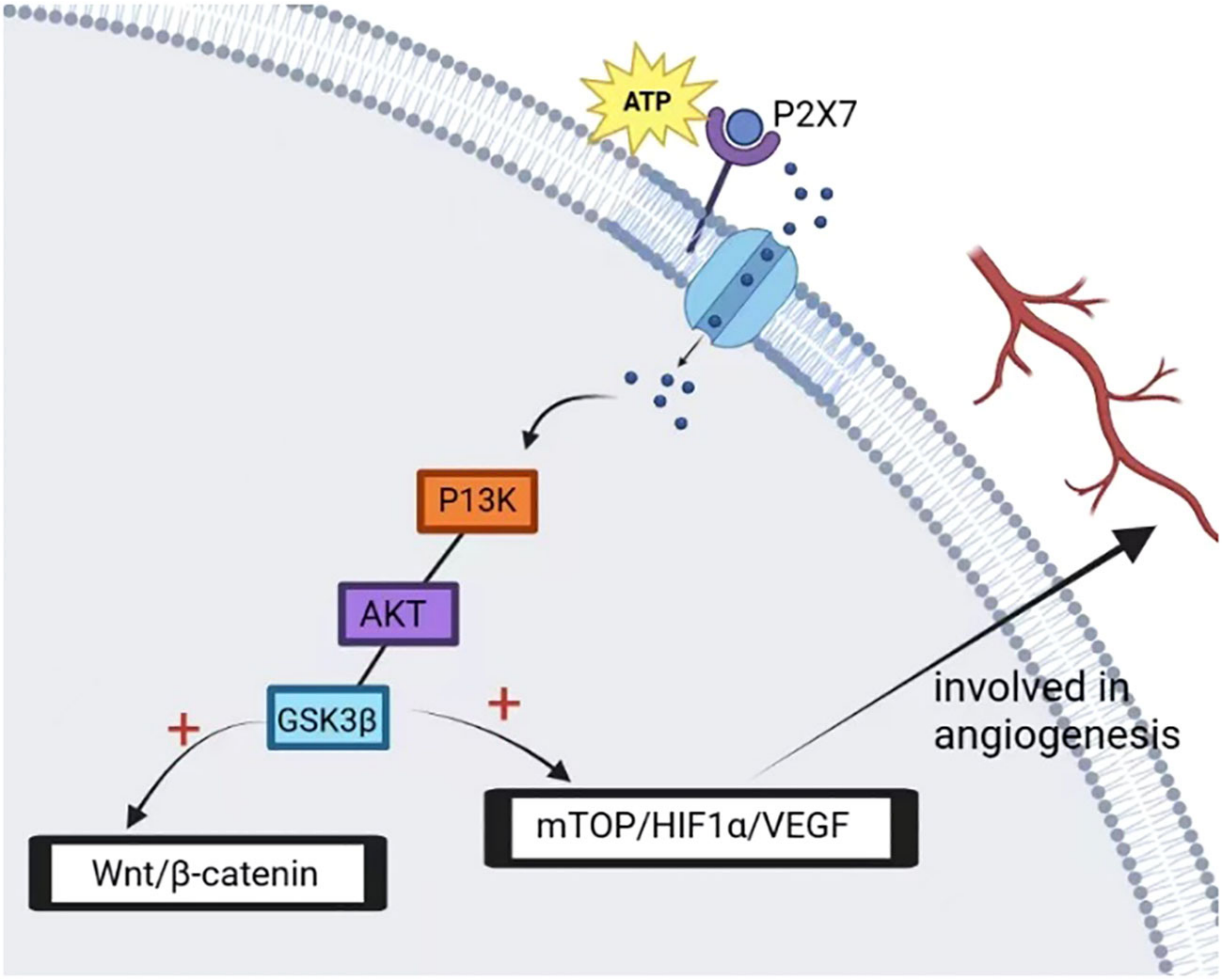
P2X7 activation mediates the opening of ion channels on the membrane, activates intracellular P13K/AKT/GSK3β signal while activating GSK3β to block glycogen synthase activity, depletes glycogen reserve and activates its downstream factor β-catenin, promotes nuclear accumulation of β-catenin, and promotes tumor cell metastasis. In addition, P2X7 activation promotes tumor angiogenesis and tumor cell growth by activating mTOP/HIF-1α/VEGF signal and upregulating the expression of HIF-1α and VEGF.

In prostate cancer cells, P2X7 plays a critical role in ATP2/BzATP-driven migration and invasion. As an ATP receptor, P2X7 activated induced P13K/AKT activity and ERK1/2 phosphorylation (87). The P13K/AKT signaling pathway is associated with the prognosis of many cancers, and the activation of the P13K/AKT signaling pathway is associated with a poor prognosis (88, 89). In lung cancer cells, the expression of P2X7 was upregulated. ATP activates P2X7, which opens ion channels in the cell membrane, thereby activating JNK and Rho kinases to regulate cell migration and induce actin remodeling. This activation also activates the HMGB1-RAGE pathway, ultimately leading to the expression of MMP2 and cytoskeletal rearrangement. These changes can ultimately affect the migration and invasion of lung cancer cells (37) (**Figure 4**). In osteosarcoma cells, BzATP stimulation can activate P2X7, leading to an increase in the activity of the P13K/AKT/GSK3β signaling pathway. The downstream signals of this pathway, including Wnt/β-catenin and mTOR/HIF1α/VEGF signaling pathways, are also activated and involved in angiogenesis (90) (**Figure 5**).

In conclusion, stimulation of the P2X7 receptor can activate the P13K/AKT signaling pathway (87, 90), decrease GSK3β activity, activate Wnt/β-catenin, increase VEGF level (90), activate JNK and Rho, and increase MMP2 expression (37), all of which are involved in tumor cell migration and invasion.

## P2X7 and epithelial–mesenchymal transition

Previous studies have shown that EMT plays an important role in the mechanism of tumor metastasis. As a receptor for ATP, the downstream pathway activated by ATP-activated P2X7 has been shown to be associated with the expression of EMT-related molecules.

During EMT, several molecular changes occur in cells, which change cell morphology and migration characteristics, and contribute to the enhanced migration and invasion ability of tumors. Basal epithelial cells lose their “epithelial phenotype” and downregulate epithelial markers while upregulating the expression of mesenchymal markers (91).

Friedl and Wolf have further classified cell migration morphology into categories of “single cell movement” or “collective cell movement” (92). Single-cell movement refers to the process by which a single cell, which has undergone a complete EMT, invades the surrounding stroma in strands and sheets. These cells lose their epithelial characteristics and acquire mesenchymal properties, allowing them to migrate and invade surrounding tissues. In contrast, collective cell movement involves a group of cells that have partially undergone EMT or are incomplete in EMT. These cells lose some of their adhesion yet keep their homotypic cell-to-cell adhesions, allowing them to break off from the whole tumor as a collective unit and invade surrounding tissues (82). Studies have indicated that cells that migrate through “collective cellular motility” are often found in lymphatic vessels to disseminate (91).

However, through reading a large number of kinds of literature, we conclude that P2X7 is related to the EMT process in different tumor invasion and metastasis processes. In prostate cancer (87), lung cancer (37), osteosarcoma (90), breast cancer (93), and gastric cancer (94), P2X7 is involved in the ATP-regulated expression process of EMT-related expression marker mRNA. Activation of P2X7 by ATP triggers downstream signaling pathways that mediate EMT, leading to the upregulation of mesenchymal phenotypic markers and the downregulation of epithelial phenotypic markers. This process is characterized by changes in cell morphology and migration characteristics and is mediated by EMT-induced transcription factors such as Snail.

## Conclusion

The pathological mechanism of tumor development is complex, and its prevention and treatment have been a current difficulty, involving the participation of different molecular substances. The tumor microenvironment is the environment in which tumors survive, and tumor cells can alter the microenvironment to better adapt to their progression. An increasing number of studies affirm the role of P2X7 in tumor progression, including gastric cancer. Activation of P2X7 directly regulates tumor growth and metastasis and indirectly regulates tumor cell progression by mediating the immune environment. In most tumor studies, including gastric cancer, P2X7 activation promoted tumor progression, while the use of P2X7 antagonists inhibited tumor growth and metastasis. In conclusion, the P2X7 may serve as a favorable molecular target for tumor prediction.

## Author contributions

W-JZ: Conceptualization, Data curation, Funding acquisition, Writing – original draft, Writing – review & editing. J-XL: Formal analysis, Methodology, Writing – original draft. G-PZ: Writing – review & editing, Formal analysis. Y-YL: Data curation, Formal analysis, Software, Writing – original draft. F-QZ: Writing – original draft, Data curation. H-JH: Writing – original draft, Data curation.
